# Effects of Perceived Neighbourhood Environments on Self-Rated Health among Community-Dwelling Older Chinese

**DOI:** 10.3390/ijerph14060614

**Published:** 2017-06-07

**Authors:** Moses Wong, Ruby Yu, Jean Woo

**Affiliations:** 1 Department of Medicine and Therapeutics, The Chinese University of Hong Kong, Hong Kong, China; moseswong@cuhk.edu.hk (M.W.); jeanwoowong@cuhk.edu.hk (J.W.); 2 CUHK Jockey Club Institute of Ageing, The Chinese University of Hong Kong, Hong Kong, China

**Keywords:** age-friendly cities, perceived neighbourhood environments, self-rated health, older Chinese, Hong Kong

## Abstract

In response to the growing number of older people living in cities, the World Health Organization (WHO) introduced the concept of “Age-Friendly Cities” (AFC) to guide the way in designing physical and social environments to encourage active ageing. Limited research has studied the effects of neighbourhood age-friendliness on elderly health outcomes. Using the example of a highly urbanized city in Asia, this study examined the effects of perceived age-friendliness of neighbourhood environments on self-rated health (SRH) among community-dwelling older Chinese. A multi-stage sampling method was used to collect views of community-dwelling older people from two local districts of Hong Kong. A structured questionnaire covering the WHO’s eight AFC domains was developed to collect information on the perceived neighbourhood environments, SRH and individual characteristics. Age-friendliness of neighbourhood was assessed by mean scores of AFC domains, which was used to predict SRH with adjustment for individual and objective neighbourhood characteristics. Furthermore, 719 respondents aged ≥60 years completed the questionnaire, of which 44.5% reported good SRH. Independent of individual and objective neighbourhood characteristics, multiple logistics regressions showed that higher satisfaction on outdoor spaces and buildings, transportation, housing, social participation, and respect and social inclusion was significantly associated with increased odds of reporting good SRH by more than 20% (*p* < 0.05). Individuals aged 70–79 years, being female, lower education and residents of public or subsidized housing were less likely to report good SRH, after controlling for individual and neighbourhood characteristics. In addition to age, gender, education and housing type, AFC environments have important contributive influence on SRH, after controlling for individual and objective neighbourhood characteristics.

## 1. Introduction

Many countries and cities worldwide are experiencing the ageing of population and urbanization. The global population of older persons aged 60 years and over reached 0.9 billion in 2015 and will grow further to nearly 2.1 billion by 2050, according to the United Nations predictions, representing a growth of the share of older persons from 12.5% to 20% in the total population [1]. Faster growth in proportion of older people has been seen in urban areas, as a result of an accelerating urbanization potentially attributable to the socio-economic developments and more favourable living circumstances in cities [1]. Hong Kong is a small but highly urbanized Special Administrative Region of China (HKSAR). In 2015, older persons aged 60 years and over accounted for 21.7% of the total population, compared to 14.8% in 2000. This proportion will further increase to 40.9% by 2050, making Hong Kong the sixth oldest territory in the world, with a median age reaching 52.7 years [1]. Hong Kong has also overtaken the world’s leading life expectancy in 2016, with average lifespans of 87.3 years for women and 81.2 year for men [2].

However, the World Report on Ageing and Health has indicated little evidence to show added years to life are effectively resulting in better health among older people [3]. A societal framework has been proposed by the World Health Organization (WHO), in which creating age-friendly cities (AFC) has been identified as one of the key priority areas for action [4].

The concept of AFC was first initiated by the WHO, with the aim to optimize opportunities for older people in terms of health, participation, security and quality of life [5]. The AFC movement commenced with a focus group study in 2006, with the participation of older people from 33 cities worldwide to identify major elements of AFC [6]. Evidence was summarized in a guide and a checklist published subsequently, in which the essential AFC features were grouped under eight key domains of urban life that encompass physical, social and service environments, including outdoor spaces and buildings, transportation, housing, social participation, respect and social inclusion, civic participation and employment, communication and information, and community support and health services [6,7]. Cities around the world were encouraged to design environments responsive to changing aspirations of older people, many of them adopted approaches and action plans specific to contexts [8,9]. With the formation of the WHO Global Network for Age-Friendly Cities and Communities, worldwide cities can exchange and learn from experiences of each other.

In 2015, the WHO Centre for Health development (the Kobe Centre) set forth a framework with a set of core and supplementary indicators to be adapted to the local context, for assessing and monitoring progress in improving age-friendly urban environments, where health and well-being were suggested as measures of the impact of age-friendly efforts [10].

Elderly people spend most of the time in their neighbourhood and their health is more sensitive to their living environments [11]. Neighourhood features have been associated with physical and mental health outcomes, even after controlling for individual characteristics such as physical activity [12], physical functioning [13], self-rated health (SRH) [14], quality of life [15], cognitive decline and depressive symptoms [16,17]. Urban built environments such as street connectivity, street and traffic safety have been associated with mobility level among older adults [18]. A recent study in Hong Kong also found that neighbourhood environmental features such as accessibility, density, personal and traffic safety and neighbourhood aesthetics were associated with an increased level of physical activity among older adults in Hong Kong [19]. Social environment was also associated with physical and mental health of older adults. Features such as social participations, helpfulness of neighbours and sense of belonging were associated with SRH, whereas mutual trust, helpfulness of neighbours and sense of belonging were associated with depression [20].

SRH has been widely used as a robust and reliable measure of self-perceived overall health condition [21,22], and has high predictability to functional disability, morbidity and mortality, especially among the older populations [23,24]. SRH of the older people has also been associated with neighbourhood environments, including physical environment such as open spaces, walkability, and air quality [25,26,27,28]; accessibility to transport options [28,29]; affordable, comfortable and well-maintained housing [27,28]; opportunities for social participation [25,30]; social cohesion and trust of neighbours [25,30,31,32,33]; opportunities for community engagement and access to local officials and local affairs [28,29,31,32]; access to and satisfaction with healthcare and amenities [28,31,32,33,34]. These associations were independent of socio-economic status (SES), lifestyle such as the level of exercise, and psychosocial factors such as loneliness, depression and stress [25,28].

In Hong Kong, the SRH of the elderly has been associated with social support and financial sufficiency [35,36]. As far as the living environment is concerned, a study of perceived neighbourhood environments in a local district by older people found education, social connection, financial sufficiency and provision of facilities and services were associated with SRH [33]. A comparative study of two other districts of Hong Kong in 2011 and 2012 found that lower scores on age-friendly features were associated with poorer SRH [37]. However, these studies were based on convenience samples, which did not capture older people from diverse backgrounds and may introduce selection bias to analysis. The neighbourhood environments were neither specific to older people nor age-friendliness [33]. Individual characteristics were not adjusted for when analyzing the association. Given the extensive literature documenting the positive association between SRH and neighbourhood characteristics, the objective of our present study examines the perceived age-friendliness of neighbourhood environments based on the WHO AFC framework and its association with SRH among community-dwelling older Chinese in Hong Kong using recent survey data, with a representative sampling strategy and adjustment for individual and objective neighbourhood characteristics.

## 2. Methods

This study used a sub-sample of an exploratory cross-sectional survey carried out across two districts (Sha Tin and Tai Po) in Hong Kong in 2015. The survey was designed using both stratified and quota sampling methods and set out to interview 500 local residents aged 18 years and above from each district. Considering the geographical heterogeneity in terms of the location of socially vulnerable groups and socio-economic characteristics, all constituency areas (CAs) of each district were stratified according to the Social Vulnerability Index (SVI) and the predominant type of housing as proxy of SES. The components and calculation of the SVI have been described elsewhere [38]. Based on the SVI, all CAs in the districts were categorized into five SVI bands with equal interval values, i.e., Band I, SVI score <2; Band II, SVI score 2–<4; Band III, SVI score 4–<6; Band IV, SVI score 6–<8; Band V, SVI score ≥8. According to the SVI values, the CAs were classified into Bands I to IV in Sha Tin, and Bands II to V in Tai Po.

For all CAs grouped under respective SVI band, we identified the nature and characteristics of CA represented by the predominant type of housing, which was determined by the type of housing accommodating the largest number of population with reference to official statistics. For CAs within the same band, we selected 3 different CAs with the largest population living in public rental housing, subsidized home ownership housing and private permanent housing. In cases where the CAs in the band were less than 3 to represent different housing characteristics, the only CA that remained in the band was selected and the sample was drawn in proportion to the population distribution in different housing types. In total, 12 CAs in Sha Tin and 11 CAs in Tai Po were selected as sampling sites. By this approach, the prospective respondents would represent views and opinions from a wide spectrum of local residents, including the most vulnerable elders and those with different socio-economic profiles.

In each CA, convenience sampling was applied. To avoid over-sampling of particular demographic representation in the final sample, quotas were set on age and sex. Accordingly, five age strata were set to include 50 samples from aged 49 years and below, 100 from 50 to 59 years, 150 from aged 60 to 69 years, 150 from aged 70 to 79 years, and 50 from aged 80 years and above, to reflect and examine divergent views on the neighbourhood environment across ages. A sex (male-to-female) ratio of approximately 0.8 was set to match with the overall sex ratio of Hong Kong.

### 2.1. Participants and Study Population

All prospective respondents were community dwellers of Chinese origin, aged 18 years and above, normally residing in Hong Kong and able to speak and understand Cantonese at the time of participation; institutionalized persons, foreign domestic helpers and individuals who were mentally incapable to communicate were excluded. To ensure reliable information, all eligible respondents had lived in our selected sampling sites for not less than six consecutive months at the time of participation in the survey. For elders who regularly visit District Elderly Community Centres (DECCs) and Neighbourhood Elderly Centres (NECs), we tried to limit the proportion to 20% in our sample, close to the average of Hong Kong, since they may represent views considerably different from other community elders [39,40].

We included all respondents aged 60 years and above into the analysis, since people aged 60–64 years are soon to be old and sometimes act as informal caregivers to their very old parents. This segment of population provides important views and valuable experiences on how an inclusive urban environment should be designed for all ages instead of for only the aged. We aimed at understanding the general impression of society on age-friendly cities, including the young-old and soon-to-be-old.

The study protocol was approved by the Survey and Behavioral Research Ethics Committee (SBREC) of the Chinese University of Hong Kong (Ethical code: 070-15). All prospective respondents were fully informed about the procedures, in speech and in writing. Written informed consent was sought from respondents prior to the interview.

Data were mainly collected by trained research assistants via face-to-face or telephone interviews (88%); a minor proportion (12%) of the relatively literate respondents self-administered the questionnaires with assistance from trained research assistants.

### 2.2. Measurements

#### 2.2.1. Survey Instrument

A structured questionnaire was used in the survey, which consisted of two major sections. The first section sought information on the respondents’ perception of the age-friendly neighbourhood environments; the second section collected the respondents’ individual characteristics, including age, gender, marital status, educational level, type of housing, residential district, total length of time having lived in the neighbourhood, living arrangement, economic activity status, occupation, prior experience of delivering informal care to elderly, use of elderly centre services, personal monthly income, and SRH.

#### 2.2.2. Perception of the Age-Friendly Neighbourhood Environments

Respondents’ perception of the age-friendly neighbourhood environments was assessed with reference to the checklist of the essential features of age-friendly cities developed by the WHO [7]. In our study, a tailor-made version of a structured questionnaire was developed, with reference to the original checklist. We examined each of the checklist features and worded according to the local context so that local residents understand and are familiar with the checklist items being asked about. The questionnaire consisted of 53 items across the eight domains [6,7], covering physical, social and service environments, which mapped onto outdoor spaces and buildings (9 items), transportation (12 items), housing (4 items), social participation (6 items), respect and social inclusion (6 items), civic participation and employment (4 items), communication and information (6 items), and community support and health services (6 items). On each item, respondents were asked to rate the age-friendliness of their neighbourhood on a six-point Likert-type scale, ranging from “strongly disagree” (1) to “strongly agree” (6). Responses to individual items were averaged to produce a mean domain score. Mean scores of all eight domains were calculated only if over half of the domain items had valid responses (1 to 6). The reliability (Cronbach’s alpha) estimate for each domain ranged from 0.65 to 0.84. Overall age-friendly environment was examined by a mean score of all domains. The higher the score, the more age-friendly neighbourhood environment was perceived.

#### 2.2.3. Objective Neighbouthood Characteristics

Objective measures of neighbourhood environment were obtained by the Geographic Information System, which provides geo-referenced environmental characteristics at 1 km radial buffer from the place of residence of respondents. Objective measures included the area of open space, road density, commercial area, government services, and vegetation cover.

#### 2.2.4. Self-Rated Health (SRH)

SRH was assessed by a single question “How would you rate your overall health at the present time?” Respondents were asked to rate their overall health on a five-point Likert scale, on which 1 indicated “poor” and 5 indicated “excellent”.

### 2.3. Data Analysis

The study population was categorized into two SRH groups as “poor” and “good” in the analysis. Respondents who rated their health status as “poor” or “fair” were grouped as “poor”, whereas those who perceived their health as “good”, “very good” or “excellent” were grouped as “good”. Bivariate analyses were used to explore the differences in individual characteristics between the SRH groups. Significant variables in the bivariate analysis were entered into the multiple logistic regressions. The presence of multicollinearity was examined using the variance inflation factor (VIF); and the presence of multicollinearity was defined as VIF >2 [41]. In our study, VIF >2 was not observed among any individual characteristics, but values >2 were observed among some of the eight AFC domains. Consequently, the mean scores of the eight age-friendly domains and the overall score of all eight domains were fitted separately into the regressions, to examine the associations between the perceived age-friendly neighbourhood environment and SRH with adjustment for individual and objective neighbourhood characteristics. Nine models were developed accordingly. Poor SRH was selected as the reference category in all logistic regression models. All statistical procedures were carried out using the Window-based SPSS Statistical Package (version 21.0; SPSS, Chicago, IL, USA), where a significant level at 5% was adopted for all statistical tests.

## 3. Results

Of the 719 respondents aged 60 years and above, the mean age was 71.6 ± 7.5 years (range 60 to 96 years). Over half of the respondents were female (54.7%). The majority was married (73.0%), living in public rental or subsidized home ownership housing (79.5%) with family or friends (85.8%). Over half had education at primary level or below (53.7%). Well over half were retirees (76.5%) and had monthly personal income <HK$10,000 (83.2%). Furthermore, 41.7% of the respondents had used services or had joined activities provided by elderly community centres in the last three months. Elder community centre users were over-represented in the study population, since the sampling strategy only controlled for the number of respondents recruited directly from elderly centres; there were respondents recruited at other community settings who were also users/members. In terms of SRH, less than half (44.5%) rated their overall health as good.

Table 1 shows the associations between SRH and the individual characteristics of the respondents. The SRH of older people in Hong Kong was significantly associated with individual characteristics such as age (*p* = 0.014), gender (*p* < 0.001), marital status (*p* = 0.004), educational level (*p* < 0.001), type of housing (*p* < 0.001), economic activity status (*p* = 0.002), use of services of elderly community centres (*p* = 0.004), and monthly personal income (*p* = 0.011). These significant variables were entered into the multiple logistic regressions as covariates.

Table 2 shows the mean scores of AFC items and domains in Sha Tin and Tai Po. Mean scores varied across the right domains, from (i) outdoor spaces and buildings (4.25 ± 0.75); (ii) transportation (4.38 ± 0.72); (iii) housing (3.79 ± 1.03); (iv) social participation (4.07 ± 1.07); (v) respect and social inclusion (3.87 ± 0.98); (vi) civic participation and employment (3.61 ± 1.14); (vii) communication and information (3.95 ± 0.90); to (viii) community and health services (3.65 ± 0.90). The mean itemized scores varied from the affordability of public transport (highest rated item: 4.95 ± 1.08) to availability and accessibility to burial sites (lowest rated item: 2.43 ± 1.42).

Results of the multiple logistic regressions are shown in Table 3. Perceived age-friendliness of the built and social neighbourhood environments was also associated with increased odds of reporting good SRH by more than 20% (*p* < 0.05), after controlling for individual and objective neighbourhood characteristics. The likelihood of reporting good health was positively and significantly associated with higher mean scores on five age-friendly domains, including outdoor spaces and buildings (Adjusted odd ratio (AOR) = 1.331; 95% CI = 1.056–1.677), transportation (AOR = 1.313; 95% CI = 1.030–1.675), housing (AOR = 1.270; 95% CI = 1.074–1.502), social participation (AOR = 1.284; 95% CI = 1.086–1.519), and respect and social inclusion (AOR = 1.215; 95% CI = 1.020–1.447). Higher mean score on community and health services domain showed positive association with SRH, but did not reach statistical significance (AOR = 1.153; 95% CI = 0.956–1.391).

When the mean score of the overall age-friendly domains was analyzed, likelihood of reporting good health was also positively associated with higher mean overall scores (AOR = 1.374; 95% CI = 1.076–1.753).

Comparing between the SRH groups, our findings reveal that those reporting good SRH were more satisfied with cleanliness, adequacy, maintenance of outdoor spaces (outdoor spaces and buildings domain), coverage and reliability of public transport (transportation domain), interior space and home modification options (housing domain) and affordable social activities at a variety of venues (social participation domain) than those reporting poor health (data not shown).

Among all objective environmental characteristics, only vegetation cover at 1 km radial buffer around the respondents’ domicile was found to be positively associated with the increased odds of reporting good SRH by four to five times (Table 3).

Individual characteristics, including age, gender, educational level and type of housing were significantly associated with the likelihood of reporting good SRH. Except for outdoor spaces and buildings, individuals aged 70–79 years were less likely to report good health compared to those aged 80 years and above, by 40%. Regardless of the eight age-friendly domains being considered, men were consistently more likely to report good SRH compared with women, adjusted for individual characteristics and neighbourhood environments. The AOR was 1.822 (95% CI 1.223–2.715) when outdoor spaces and buildings domain was fitted into the regression, whereas the AORs were 1.775 (95% CI 1.192–2.645) in the transportation model, 1.767 (95% CI 1.184–2.639) in the housing model, 1.792 (95% CI 1.201–2.674) in the social participation model, 1.803 (95% CI 1.211–2.686) in the respect and social inclusion model, 1.770 (95% CI 1.190–2.634) in the civic participation and employment model, 1.790 (95% CI 1.203–2.663) in the information and communication model, and 1.779 (95% CI 1.196–2.648) in the community support and health services model, respectively.

Older people with lower educational level were less likely to report good SRH compared with their counterparts having post-secondary education. The AOR of reporting good health among those who had primary education and below was 0.245 (95% CI 0.121–0.496) when outdoor spaces and buildings domain was fitted into the regression, whereas the AORs were 0.251 (95% CI 0.124–0.506) in the transportation model, 0.247 (95% CI 0.122–0.500) in the housing model, 0.242 (95% CI 0.119–0.489) in the social participation model, 0.245 (95% CI 0.121–0.496) in the respect and social inclusion model, 0.252 (95% CI 0.125–0.508) in the civic participation and employment model, 0.256 (95% CI 0.127–0.517) in the information and communication model, and 0.254 (95% CI 0.126–0.513) in community support and health services model, respectively.

Older people living in public rental or subsidized home ownership housing were less likely to report good SRH compared with their counterparts living in private permanent housing. The AOR of reporting good health among those living in public rental housing was 0.414 (95% CI 0.246–0.696) when outdoor spaces and buildings domain was fitted into the regression, whereas the AORs were 0.412 (95% CI 0.244–0.694) in the transportation model, 0.388 (95% CI 0.229–0.656) in the housing model, 0.408 (95% CI 0.242–0.687) in the social participation model, 0.419 (95% CI 0.249–0.703) in the respect and social inclusion model, 0.448 (95% CI 0.267–0.752) in the civic participation and employment model, 0.448 (95% CI 0.268–0.751) in the information and communication model, and 0.442 (95% CI 0.250–0.714) in the community support and health services model, respectively. 

## 4. Discussion

### 4.1. Key Findings 

Creating age-friendly environments to promote elderly health and well-being has received increasing interest from academic and public policy fronts. To date, however, there has been little research examining age-friendly environments in relation to elder outcomes in Hong Kong. Our study examined the perceived age-friendliness of neighbourhood environments based on the WHO AFC framework and its association with SRH among community-dwelling older Chinese in Hong Kong. We found that higher satisfaction on five age-friendly domains, namely, outdoor spaces and building, transportation, housing, social participation, and respect and social inclusion, were associated with greater odds of reporting good SRH, after adjusting for individual and objective neighbourhood characteristics.

In our study, perceived age-friendliness of built and social environments of the neighbourhood was associated with the likelihood of reporting good health. Prior research has identified cosmetic features, crowdedness, air pollution, noise, housing condition, accessibility to transport and open space as physical environmental factors associated with health [25,26,27,28,29,32], whereas opportunities for social participation and friendliness of community members are social environmental factors [25,30,31,32,33]. Using surveys of a large sample of older persons in the UK, Poortinga et al. examined the perceptions of neighbourhood environment and the association with SRH in a multilevel analysis. It was found that poor access to amenities and poor neighbourhood quality were associated with increased odds of reporting poor health, and the association remained significant even after controlling for individual characteristics and neighbourhood deprivation [32]. Similar findings were documented in an analysis of age-friendly environment in the US, where older persons perceived neighbourhood problems such as safety, crime and housing problems were associated with poorer SRH [31]. Green outdoor environment was found to have a positive impact on SRH by experiences of being away and fascination and increased outdoor visits [42,43]. Sufficient and decent outdoor space, and a safe neighborhood may influence health by having an impact on an active lifestyle. Stronegger et al. showed that higher satisfaction on local infrastructure was associated with higher levels of physical activity for transport (cycling) [44], whereas a recent study in Hong Kong has found that pedestrian infrastructure, street connectivity, physical barriers and crowdedness were associated with physical activity level in terms of walking duration and frequency [19]. These observations are confirmed in our findings, in which higher satisfactions on outdoor spaces and transportation domains were associated with more than 30% of increased odds of reporting good health adjusted for individual characteristics and objective environments. These observed AORs were slightly stronger than housing (27%), social participation (28.4%) and respect and social inclusion (21.5%).

In terms of social environment, our study showed that social participation, and respect and social inclusion were associated with higher odds of reporting good health. Higher rate of social participation, social support and exchange with neighbours were found to have a positive impact on SRH [31,33,45], whereas trust and helpfulness of community members were also associated with less reported depressive symptoms [20]. Stronegger et al. showed that greater satisfaction on the overall social environment was associated with higher odds of reporting good health and leisure time physical activity. SRH was independently associated with leisure time physical activity and satisfaction on environmental quality [44]. A good social environment is an ideal habitat for nurturing social capital and sense of belonging. An inclusive and mutually respectful environment is important for exchanges and mutual understanding. 

Interestingly, community support and health services showed a positive but insignificant association with SRH. The AFC aspects of this domain focus on provision, accessibility and affordability of services, the satisfaction level of which may not be necessarily associated with health status and actual health service usage. Moreover, positive perception on community support and health services may be determined by factors other than health status and usage experience, including physical distance and economic barriers to accessing them.

Perceived neighbourhood environments have been analyzed in addition to objective environment in the studies of SRH [28], physical activity, quality of life and depression among older adults [46]. It was found that objective neighbourhood SES was significantly associated with SRH, but the effect is substantially explained by individual SES and perceptions of neighbourhood, yet a significant effect of perceived neighbourhood quality on SRH was found even after controlling for individual characteristics and neighbourhood SES. Our study also included some objectively measured environmental features, and the result showed that vegetation cover was associated with four- to five-fold increased likelihood of reporting good health among older persons, after controlling for personal characteristics and age-friendly neghbourhood perceptions. Previous studies have shown that green space was associated with positive physical and mental health outcomes [47,48], by stress reduction and increased frequency of outdoor visits and physical activity. Information on lifestyle is essential to understanding how vegetation influences SRH.

However, interpretation of the possible health effects of these environmental factors may not be straightforward, since the environmental effects substantially attenuated after controlling for individual or neighbourhood SES [32,49]. Moreover, environmental effects on health were more explained by perceived neighbourhood quality rather than objective measures, and part of the effect was mediated by psychological factors such as loneliness, depression, hostility and stress [28].

Our results showed that individuals aged 70–79 years were less likely to report good health than the old-old. Some studies have reported better health ratings among the old-old than among the young-old [50,51]. The pattern could possibly be explained by way and time that they cope with disease and disability, the process of ageing, the selective survivorship and cohort differences.

Our findings are consistent with previous research demonstrating gender difference of health status [52,53]. WHO suggested that gender differences in health are the result of both biological and social factors [54], including different risk exposure from employment, and risk taking behaviors such as smoking and alcohol consumption [55]. In our study, women appeared to be less likely to report good SRH than men, after controlling for individual and neighborhood characteristics. Women tended to report more somatic complaints in Hong Kong [56,57]. Taking use of health services as the outcome measure, women in Hong Kong were more likely to visit Western medical practitioners but not necessarily incur hospitalization service, after adjusting for predisposing factors, enabling factors and need factors. Need factors such as presence of a doctor-diagnosed chronic illness, SRH status, short-form-12 physical and mental component scores were the most significant determinant of increased health care utilization among women in Hong Kong, reflecting that the gender difference of health in Hong Kong is both objective and subjective [58].

A strong positive association was observed in our study between education and SRH after adjusting for individual and neighbourhood characteristics, which is consistent with existing literature. Higher education is often associated with having a fullfilling job and higher income, having higher healthy literacy, and a greater sense of controlling their behaviours and developing a healthier lifestyle [59]. Yanfound that older person tended to rated health more positively [33]; whereas Xu et al. reported the fact that most of the elders in China had less education than younger generations [53]. Therefore, the influence of age should be adjusted for when examining the association between education and health.

Our study also found an association between SRH and the type of housing. Hong Kong currently operates the largest public housing system among cities in the capitalist economies. The government-led public housing programme offers homes to 47% of the population in the form of public rental flats and subsidized home ownership scheme [60]. A recent study investigated the association between the provision of public housing in Hong Kong in relation to the variation of premature mortality across the city, and found that premature mortality risk was negatively associated with the proportion of residents residing in public housing, after controlling for neighbourhood deprivation, access to local amenities, concentration of elder residents and other neighbourhood characteristics [49]. However, the study did not investigate or compare the relative risk across different types of housing, and the use of premature mortality may not represent the overall health among the older population. 

Our findings suggested that residents living in public rental and subsidized home ownership flats were less likely to report good SRH than their counterparts living in private housing, consistent with studies carried out in Western cities [61,62]. Literature on the association between housing and health has primarily focused on the environmental or physical aspects of housing, the association between physical and tenure patterns, as well as neighbourhood characteristics [63]. It has been suggested that substandard housing conditions and poor social, physical and economic characteristics of the neighbourhood of social or public housing were often associated with negative health effects on residents [64,65]. A study examining the health impact of relocation into public housing found that the majority of residents were already ill before moving into the public housing estate [66], suggesting the possibility of health selection by which the social allocation system filters people with poor health into public housing.

### 4.2. Limitations

Several limitations of the present study should be addressed. The cross-sectional design demonstrated the associations but is limited to elucidating the mechanism and causality of the association. Secondly, the study population was not recruited from a random sampling strategy and only included community-dwelling older people, which may be a source of selection bias by omitting those who were home-bound and have limited access to outdoor environments. The result may not be generalizable to the Hong Kong general population. Moreover, the questionnaire respondents were likely to represent a more active group of older people, whose views have the potential for recall bias leading to a more favourable general opinion on the neighbourhood environment. However, this proportion of which was carefully considered and they were not oversampled in our study population. To minimize the bias arising from the sampling methods, we have chosen the sampling criteria to achieve the most representative sample possible, to cover a wide spectrum of the older people who were at different levels of social vulnerability risk, socio-economic background, demographic structure and social participation. Finally, when examining the effects of perceived neighbourhood environments on SRH, there may be other variables such as disability, number of acute and chronic diseases and depression affecting the SRH of an individual, but were not included in the present study. In this study, we have adjusted estimates for individual characteristics that influenced the association between environment and SRH. Future research should include individuals’ medical history, lifestyle factors, and psychosocial measures that may interact with the association between environment and health. Other elder outcome measures such as functional ability, depression, loneliness, well-being and quality of life could also be considered to evaluate the overall benefits of age-friendly environments to older people. Since data was collected from a total of 23 CAs nested in two districts of Hong Kong, multi-level analysis was impractical. Structural Equation Model or Multiple Mediation Analysis could be considered as methodology to identify underlying mechanisms on how age-friendly neighbourhood affects health outcomes. Despite these limitations, our study provided an updated examination of the perceived age-friendly neighbourhood environments by using the WHO framework and its effects on SRH among community-dwelling older Chinese aged 60 years and above in Hong Kong. We have also addressed some of the shortcomings of the convenience sample. Our study has also controlled for individual characteristics when examining the effect of neighbourhood environments on SRH, while similar research did not adjust for confounding variables. The results of this study can inform future research work in the local or other highly urbanized and fast aging cities in Asia.

### 4.3. Future Directions

The concept of an age-friendly city is to create the environment that encourages active ageing to enhance the quality of life as people age. In practical terms, an age-friendly neighbourhood should adapt its structures and services to be accessible to and inclusive of older people with varying needs and capacities. The Government of the Hong Kong SAR has recently promulgated a review on territorial strategy to guide planning of built environment of Hong Kong beyond 2030 (“Hong Kong 2030+”) [67]. Creating inclusive and supportive features in a liveable high-density city has been set as one of the building blocks, according to which an age-friendly environment will be promoted to encourage active ageing, ageing in place and intergenerational support. The vision indicates that this is a promising area for future research, particularly since few age-friendly environment frameworks in the region call attention to the potential effects of these features on elderly population. Future research should also examine the potential variations in the effects of age-friendly environment on health across intra-city contexts as well as consider other elder outcomes potentially associated with age-friendly environment.

## 5. Conclusions

This study is among the first to examine association between age-friendly environments on elder outcomes, in this case SRH, and provides a foundation for future research. It supports previous findings regarding effects of physical and social environments on elder health, and suggests the importance of including both physical and social environmental characteristics in future studies of SRH. 

## Figures and Tables

**Table 1:** Bivariate associations between self-rated health and independent variables.

Individual Characteristics		Poor/Fair (*n* = 399)		Good/Very Good/Excellent (*n* = 320)		*p*
		*n*	(%)	*n*	(%)	
Age (years)	60–69	150	(37.6)	155	(48.4)	0.014
	70–79	182	(45.6)	122	(38.1)	
	≥80	67	(16.8)	43	(13.4)	
Gender	Men	140	(35.1)	186	(58.1)	<0.001
	Women	259	(64.9)	134	(41.9)	
Marital status ^a,b^	Currently married	274	(69.0)	251	(78.7)	0.004
	Currently not married	123	(31.0)	68	(21.3)	
Educational level	Primary and below	255	(63.9)	131	(40.9)	<0.001
	Secondary	129	(32.3)	140	(43.8)	
	Post-secondary	15	(3.8)	49	(15.3)	
Type of housing ^b^	Public rental	126	(31.6)	63	(19.7)	<0.001
	Subsidized home ownership	176	(44.1)	130	(40.6)	
	Private permanent	92	(23.1)	125	(39.1)	
	Temporary	5	(1.3)	2	(0.6)	
Living arrangement ^a^	Living alone	58	(14.5)	44	(13.8)	0.764
	Not living alone	341	(85.5)	276	(86.2)	
Economic activity status ^a^	Employed	22	(5.5)	32	(10.0)	0.002
	Retired	299	(74.9)	251	(78.4)	
	Others	78	(19.5)	37	(11.6)	
Prior experience of delivering informal care to elderly ^b^	No	180	(45.3)	152	(47.5)	0.564
	Yes	217	(54.7)	168	(52.5)	
Use of elderly centre services ^b^	No	213	(53.5)	205	(64.3)	0.004
	Yes	185	(46.5)	114	(35.7)	
Monthly personal income ^b^	<10,000	345	(87.6)	253	(80.6)	0.011
	≥10,000	49	(12.4)	61	(19.4)	
Residential district	Sha Tin	209	(52.4)	155	(48.4)	0.293
	Tai Po	190	(47.6)	165	(51.6)	
Length of residence in current neighbourhood (years) (mean, ±SD)		23.3	±12.1	22.7	±13.1	0.537

^a^ Marital status was categorized into two groups: “Currently married” and “Currently not married”, the latter included those who were never married, widowed, separated and divorced; living arrangement was categorized into two groups: “Living alone” and “Not living alone”, the latter included those living with parent(s), spouse and/or child(ren), or other members; economic activity status was categorized into three groups: “Employed”, “Retired” and “Others”, the latter included unemployed persons, students and home-makers; ^b^ Data were missing on marital status (*n* = 3), prior experience of delivering informal care to elderly (*n* = 2), use of community centre services (*n* = 2), monthly personal income (*n* = 11). Respondents living in temporary housing (*n* = 7) were excluded. Percentages may not total 100 due to rounding.

**Table 2:** Mean scores of the WHO AFC items and domains in Sha Tin and Tai Po.

WHO AFC Items and Domains	*n*	Mean	SD
Item A1: Cleanliness	719	4.61	1.00
Item A2: Adequacy, Maintenance and Safety	718	4.53	1.08
Item A3: Drivers’ Attitude at Pedestrian Crossings	718	4.29	1.17
Item A4: Cycling Lanes	718	4.41	1.32
Item A5: Outdoor Lighting and Safety	718	4.50	1.10
Item A6: Accessibility of Commercial Services	719	4.49	1.26
Item A7: Arrangement of Special Customer Service to Persons in Need	718	3.31	1.60
Item A8: Building Facilities	718	4.15	1.34
Item A9: Public Washrooms	719	4.01	1.38
***Domain 1: Outdoor Spaces and Buildings***	***719***	***4.25***	***0.75***
Item B10: Traffic Flow	719	4.67	0.93
Item B11: Coverage of Public Transport Network	719	4.85	1.01
Item B12: Affordability of Public Transport	718	4.95	1.08
Item B13: Reliability of Public Transport	717	4.35	1.22
Item B14: Public Transport Information	716	4.06	1.39
Item B15: Condition of Public Transport Vehicles	718	4.62	1.06
Item B16: Specialized Transportation for disabled people	713	3.82	1.57
Item B17: Transport Stops and Stations	718	4.58	1.07
Item B18: Behaviour of Public Transport Drivers	718	4.58	1.11
Item B19: Alternative Transport in Less Accessible Areas	713	3.56	1.56
Item B20: Taxi	712	3.75	1.52
Item B21: Roads	717	4.71	0.97
***Domain 2: Transportation***	***718***	***4.38***	***0.72***
Item C22: Sufficient and Affordable Housing	717	4.01	1.48
Item C23: Interior Spaces and Level Surfaces of Housing	719	4.58	1.15
Item C24: Home Modification Options and Supplies	715	3.29	1.61
Item C25: Housing for Frail and Disabled Elders	711	3.28	1.61
***Domain 3: Housing***	***717***	***3.79***	***1.03***
Item D26: Mode of Participation	719	4.41	1.31
Item D27: Participation Costs	716	4.34	1.42
Item D28: Information about Activities and Events	718	4.05	1.46
Item D29: Variety of Activities	717	4.13	1.38
Item D30: Variety of Venues for Elders’ Gatherings	718	4.07	1.45
Item D31: Outreach Services to People at Risk of Social Isolation	711	3.43	1.60
***Domain 4: Social Participation***	***719***	***4.07***	***1.07***
Item E32: Consultation from Different Services	719	3.49	1.58
Item E33: Variety of Services and Goods	715	3.59	1.41
Item E34: Manner of Service Staff	719	4.55	1.14
Item E35: School as Platform for Intergeneration Exchange	714	3.29	1.62
Item E36: Social Recognition	716	4.22	1.31
Item E37: Visibility and Media Depiction	713	4.08	1.29
***Domain 5: Respect and Social Inclusion***	***719***	***3.87***	***0.98***
Item F38: Options for Older Volunteers	715	3.80	1.54
Item F39: Promote Qualities of Older Employees	711	3.87	1.47
Item F40: Paid Work Opportunities for Older People	715	3.35	1.58
Item F41: Age Discrimination	708	3.44	1.53
***Domain 6: Civic Participation and Employment***	***718***	***3.61***	***1.14***
Item G42: Effective Communication System	716	4.34	1.22
Item G43: Information and Broadcasts of Interest to Elders	717	3.85	1.42
Item G44: Information to Isolated Individuals	705	3.68	1.42
Item G45: Electronic Devices and Equipment	718	4.38	1.19
Item G46: Automated Telephone Answering Services	714	3.61	1.55
Item G47: Access to Computers and Internet	712	3.88	1.60
***Domain 7: Communication and Information***	***718***	***3.95***	***0.90***
Item H48: Adequacy of Health and Community Support Services	719	4.18	1.32
Item H49: Home Care Services	718	3.57	1.53
Item H50: Proximity between Old Age Homes and Services	719	4.12	1.35
Item H51: Economic barriers to Health and Community Support Services	718	4.22	1.30
Item H52: Community Emergency Planning	714	3.34	1.55
Item H53: Burial Sites	715	2.43	1.42
***Domain 8: Community Support and Health Services***	***719***	***3.65***	***0.90***

**Table 3:** Multiple logistic regressions assessing significant factors associated with self-rated health.

Variables	Good vs. Poor		Good vs. Poor		Good vs. Poor		Good vs. Poor		Good vs. Poor		Good vs. Poor		Good vs. Poor		Good vs. Poor		Good vs. Poor	
	B	AOR (95% CI)	B	AOR (95% CI)	B	AOR (95% CI)	B	AOR (95% CI)	B	AOR (95% CI)	B	AOR (95% CI)	B	AOR (95% CI)	B	AOR (95% CI)	B	AOR (95% CI)
*N^†^*	688		687		686		688		688		687		687		688		688	
*Age (years)*																		
60–69																		
70–79			−0.524	0.592 (0.356, 0.986) *	−0.539	0.584 (0.351, 0.971) *	−0.532	0.587 (0.354, 0.976) *	−0.515	0.598 (0.361, 0.991) *	−0.511	0.600 (0.362, 0.994) *	−0.524	0.592 (0.356, 0.985) *	−0.514	0.598 (0.361, 0.991) *	−0.541	0.582 (0.351, 0.967) *
≥80 (ref)			0	1	0	1	0	1	0	1	0	1	0	1	0	1	0	1
*Gender*																		
Men	0.600	1.822 (1.223, 2.715) **	0.574	1.775 (1.192, 2.645) **	0.570	1.767 (1.184, 2.639) **	0.583	1.792 (1.201, 2.674) **	0.590	1.803 (1.211, 2.686) **	0.571	1.770 (1.190, 2.634) **	0.582	1.790 (1.203, 2.663) **	0.576	1.779 (1.196, 2.648) **	0.579	1.783 (1.197, 2.657) **
Women (ref)	0	1	0	1	0	1	0	1	0	1	0	1	0	1	0	1	0	1
*Marital status*																		
Currently married																		
Currently not married (ref)																		
*Educational level*																		
Primary and below	−1.408	0.245 (0.121, 0.496) ***	−1.382	0.251 (0.124, 0.506) ***	−1.399	0.247 (0.122, 0.500) ***	−1.420	0.242 (0.119, 0.489) ***	−1.405	0.245 (0.121, 0.496) ***	−1.377	0.252 (0.125, 0.508) ***	−1.362	0.256 (0.127, 0.517) ***	−1.369	0.254 (0.126, 0.513) ***	−1.395	0.248 (0.123, 0.501) ***
Secondary	−0.867	0.420 (0.212, 0.832) *	−0.875	0.417 (0.211, 0.822) *	−0.864	0.422 (0.213, 0.836) *	−0.905	0404 (0.204, 0.800) **	−0.882	0.414 (0.209, 0.819) *	−0.875	0.417 (0.212, 0.822) *	−0.863	0.422 (0.214, 0832) *	−0.853	0.426 (0.216, 0.841) *	−0.873	0.418 (0.211, 0.826) *
Post-secondary (ref)	0	1	0	1	0	1	0	1	0	1	0	1	0	1	0	1	0	1
*Type of housing*																		
Public rental	−0.882	0.414 (0.246, 0.696) **	−0.887	0.412 (0.244, 0.694) **	−0.947	0.388 (0.229, 0.656) ***	−0.897	0.408 (0.242, 0.687) ***	−0.871	0.419 (0.249, 0.703) **	−0.802	0.448 (0.267, 0.752) **	−0.802	0.448 (0.268, 0.751) **	−0.862	0.422 (0.250, 0.714) **	−0.933	0.394 (0.232, 0.667) **
Subsidized home ownership	−0.592	0.553 (0.359, 0.853) **	−0.583	0.558 (0.362, 0.860) **	−0.625	0.535 (0.346, 0.828) **	−0.595	0.551 (0.357, 0.851) **	−0.573	0.564 (0.366, 0.869) **	−0.536	0.585 (0.381, 0.898) *	−0.552	0.576 (0.374, 0.885) *	−0.566	0.568 (0.369, 0.874) **	−0.600	0.549 (0.355, 0.847) **
Private permanent (ref)	0	1	0	1	0	1	0	1	0	1	0	1	0	1	0	1	0	1
*Economic activity status*																		
Employed (ref)																		
Retired																		
Others																		
*Monthly personal income*																		
<10,000 HKD																		
≥10,000 HKD (ref)																		
*Users of elderly centre*																		
No (ref)																		
Yes																		
*Objective environment*																		
Outdoor spaces																		
Vegetation	1.673	5.326 (1.248, 22.727) *	1.536	4.645 (1.104, 19.545) *	1.506	4.507 (1.072, 18.954) *	1.617	5.037 (1.186, 21.353) *	1.527	4.605 (1.098, 19.320) *			1.456	4.289 (1.025, 17.939) *	1.435	4.202 (1.009, 17.505) *	1.598	4.943 (1.170, 20.880) *
Road area																		
Commercial area																		
Government services																		
*AFC environments*																		
Outdoor spaces and buildings	0.286	1.331 (1.056, 1.677) *																
Transportation			0.273	1.313 (1.030, 1.675) *														
Housing					0.239	1.270 (1.074, 1.502) **												
Social participation							0.250	1.284 (1.086, 1.519) **										
Respect and social inclusion									0.195	1.215 (1.020, 1.447) *								
Civic participation and employment																		
Information and communication																		
Community support and health services																		
Overall scores of age-friendly domains																	0.317	1.374 (1.076,1.753) *
Goodness-of-fit ^§^	0.638		0.296		0.901		0.785		0.601		0.249		0.299		0.612		0.580	

B, Coefficient; AOR, Adjusted odd ratio; CI, confidence interval; Figures were obtained from multiple logistic regressions, controlling for objective neighbourhood environment measures and variables that were significant in the bivariate analyses (Table 1), which included age, gender, marital status, education level, type of housing, economic activity status, monthly personal income, and user of an elderly centre. Individual and overall age-friendly environments were separately examined in the models using poor self-rated health as reference category; only significant values are shown in the table; * *p* < 0.05; ** *p* < 0.01; *** *p* < 0.001; ^†^ Number of observations included in the logistic regression models; ^§^ Goodness-of-fit statistics were obtained from Hosmer and Lemeshow tests.
